# Morph-specific protein patterns in the femoral gland secretions of a colour polymorphic lizard

**DOI:** 10.1038/s41598-019-44889-7

**Published:** 2019-06-10

**Authors:** Marco Mangiacotti, Marco Fumagalli, Maddalena Cagnone, Simona Viglio, Anna Maria Bardoni, Stefano Scali, Roberto Sacchi

**Affiliations:** 1 Department of Earth and Environmental Sciences, University of Pavia, Via Taramelli 24, 27100 Pavia, Italy; 2 Department of Biology and Biotechnologies “L.Spallanzani”, Unit of Biochemistry, University of Pavia, Via Ferrata 9, 27100 Pavia, Italy; 3 Department of Molecular Medicine, Unit of Biochemistry, University of Pavia, Via T. Taramelli 3, 27100 Pavia, Italy; 4 Museo di Storia Naturale di Milano, Corso Venezia 55, 20121 Milan, Italy

**Keywords:** Chemical ecology, Peptides, Behavioural ecology

## Abstract

Colour polymorphism occurs when two or more genetically-based colour morphs permanently coexist within an interbreeding population. Colouration is usually associated to other life-history traits (ecological, physiological, behavioural, reproductive …) of the bearer, thus being the phenotypic marker of such set of genetic features. This visual badge may be used to inform conspecifics and to drive those decision making processes which may contribute maintaining colour polymorphism under sexual selection context. The importance of such information suggests that other communication modalities should be recruited to ensure its transfer in case visual cues were insufficient. Here, for the first time, we investigated the potential role of proteins from femoral gland secretions in signalling colour morph in a polymorphic lizard. As proteins are thought to convey identity-related information, they represent the ideal cues to build up the chemical modality used to badge colour morphs. We found strong evidence for the occurrence of morph-specific protein profiles in the three main colour-morphs of the common wall lizard, which showed both qualitative and quantitative differences in protein expression. As lizards are able to detect proteins by tongue-flicking and vomeronasal organ, this result support the hypothesis that colour polymorphic lizards may use a multimodal signal to inform about colour-morph.

## Introduction

Among the most intriguing phenomena able to recursively animate the debate and to stimulate theoretical work in evolutionary biology, colour polymorphism (CP) surely occupies a good standing^1,2^. Its usually preferred definition, which somehow encloses the reason itself for the interest, is that of Huxley^3^, who slightly reformulated the original one by Ford^4^: CP occurs when two or more heritable colour morphs “*coexist in temporary or permanent balance within a single interbreeding population […] in such frequencies that the rarer cannot be due solely to mutation*”^3^. Colour is usually associated to other individual traits (physiological, morphological, ecological, reproductive, behavioural)^1,5,6^, resulting the most apparent attribute among a set of correlated ones^1,5–9^. Each morph can be viewed as an alternative combination of characters within a species, occupying a different peak in the adaptive landscape^1^. Understanding the mechanisms able to maintain (even “temporarily”) a balanced morph composition against recombination and genetic drift, which should operate in the opposite direction, has been viewed as the key for a deeper comprehension of evolutionary processes^1,5,6,10–14^.

Even if CP is generally regarded as any other polymorphism^1,3^, it intrinsically and inevitably pertains also to the sphere of animal communication^15–17^. When CP is driven by sexual selection, colour represents the visible badge of the underlying set of correlated traits^6^ and, as such, it is used to modulate the intra- and inter-specific interactions upon which CP maintenance is based^9,18^. Non-random pairing as well as morph-specific aggressiveness were often found to be the main behavioural mechanisms^6,9^, which require colour to be the intraspecific signal mediating decision-making processes^18^. In such contexts, communicating the own morph to conspecifics is advantageous to both signaller and receiver, and the morph-identity function of colour is therefore promoted and maintained^19^. Communication plays such a pivotal role in the mechanism that one could expect that other (even all) channels must be recruited to ensure its reliability and efficacy^16,20,21^. Indeed, some evidence of non-visual communication modalities matching colour morphs have been already found in orchids^22–24^, insects^25,26^, fish^27–29^, amphibians^30,31^, and lizards^32–34^. In all the above cases, the role of non-visual channel is to make the visual one more effective, ensuring that the message will be delivered when colour alone is not enough or cannot be detected^35^.

Lizards offer an ideal model to elucidate the interactions between visual and non-visual communication in association to CP. Firstly, CP is widespread and well-studied in this group^6^, and has been extensively used for theoretical works^7,10–12,36–39^. Secondly, as sexual selection and social strategies seem to play a major role in maintaining CP in lizards^12,34,39–46^, the need for an unbiased communication system is strengthened^16,18,21,47,48^. Finally, lizards have well-developed visual and chemical sensory systems, which constitute the hard-core of their social communication^11,49–58^. Notably, on the receiver side, chemoreception is powered by the vomeronasal organ associated to a forked tongue and the tongue-flicking behaviour^58–61^. On the signaller side, most lizards species have a series of specialized epidermal glands in the femoral and/or pre-cloacal region^62–64^ producing waxy secretions used to convey information about many signaller’s traits, like species^65–67^, sex^68–70^, identity^71–73^, familiarity^50,74–76^, status^77–80^, and condition^81,82^. Therefore, the chemical path comes as the ideal channel being combined to the visual modality explicitly recalled by CP.

Lizard femoral gland secretions are made of a mix of lipids and proteins^83,84^ whose relative proportion seems to vary with species considered^84–86^ and along the activity season, following androgen levels^86,87^. Unfortunately, only few data on a bunch of species are actually available^63,73^. The lipophilic fraction, which has been extensively studied, usually includes steroids, terpenes, provitamins (D and E), long chain acids, alcohols, esters, ketones, aldehydes, all being precursors, products or by-products of fat metabolism^83,88^. Given the cost they impose to the signaller, lipids have been hypothesized to honestly convey quality- and condition-related information used by conspecifics to make a decision in both intersexual (mate choice) or intrasexual interactions (male-male combats)^53^. For example, females of the well-studied lacertid lizard *Iberolacerta monticola* prefer territories marked by ergosterol-enriched scent of males with better immunity and condition^89^. Males are still able to assess fighting ability of the potential opponent based on the cholesterol level in the femoral secretions^78^. Similar evidences were also found in other lizard species^65,90–92^.

By contrast, the protein fraction is poorly known. The pioneering studies on the desert iguana (*Dipsosaurus dorsalis*) and the green iguana (*Iguana iguana*) showed that proteins could be used as signal, probably conveying identity-related information^69,76,84,87^, and support to such function has been recently confirmed for a lacertid species^93^. Combined to the expected strong relation between proteins and genes, these findings suggest that proteins may play an important role in individual recognition on a chemical basis^63,73,94^, which is a key pre-requisite in driving lizard social behaviour^80,95,96^. Since colour morph represents a genetic condition of the individual, not related to its body condition^97^, selection should promote the coevolution of: (i) an encoding system of the information about the signaller’s morph, especially in the protein fraction of the femoral gland secretions, and (ii) a decoding system of protein fraction associated to the vomeronasal organ^54^ of conspecific males or females. This would be the only way by which information may help individuals to drive behavioural choices and therefore contribute to the CP maintenance^42,98^.

To verify the hypothesis that proteins from femoral glands have the potential to convey information about colour morph, we analysed and compared the protein profiles from the three main morphs of the common wall lizard (*Podarcis muralis*)^42,99^. The ventral colouration (yellow, red/orange, and white) is genetically controlled^100^, and has been already correlated to many other traits^41,43,101–106^, even though a clear pattern has not still emerged. A potential environmental role in CP expression has been recently documented, suggesting that both natural and sexual selection may be involved in CP expression^107,108^. Nonetheless, the signal function of the ventral colouration is strongly supported by the morph assortative pairing^42,45,46^, by the morph-specific male-male interactions^109,110^, and by the lizard ability to discriminate colour morph^18^. Further, previous studies have already highlighted the occurrence of a chemical segregation of morphs^41^. Some lipophilic compounds, namely, tocopherol, are actually differentially allocated by morphs in the femoral pore secretions^32^, and 1-D electrophoretic runs performed on proteins of different populations of this species have shown an among-individuals variability in the profiles in terms of occurrence and intensity of some distinct protein bands^73^. However, the comparison and characterization of the proteins from the three main colour morphs have never been attempted. Here, differentially expressed proteins were detected and tentatively identified for the first time.

## Results

### Two-dimensional electrophoresis (2-DE)

**Figure 1:**
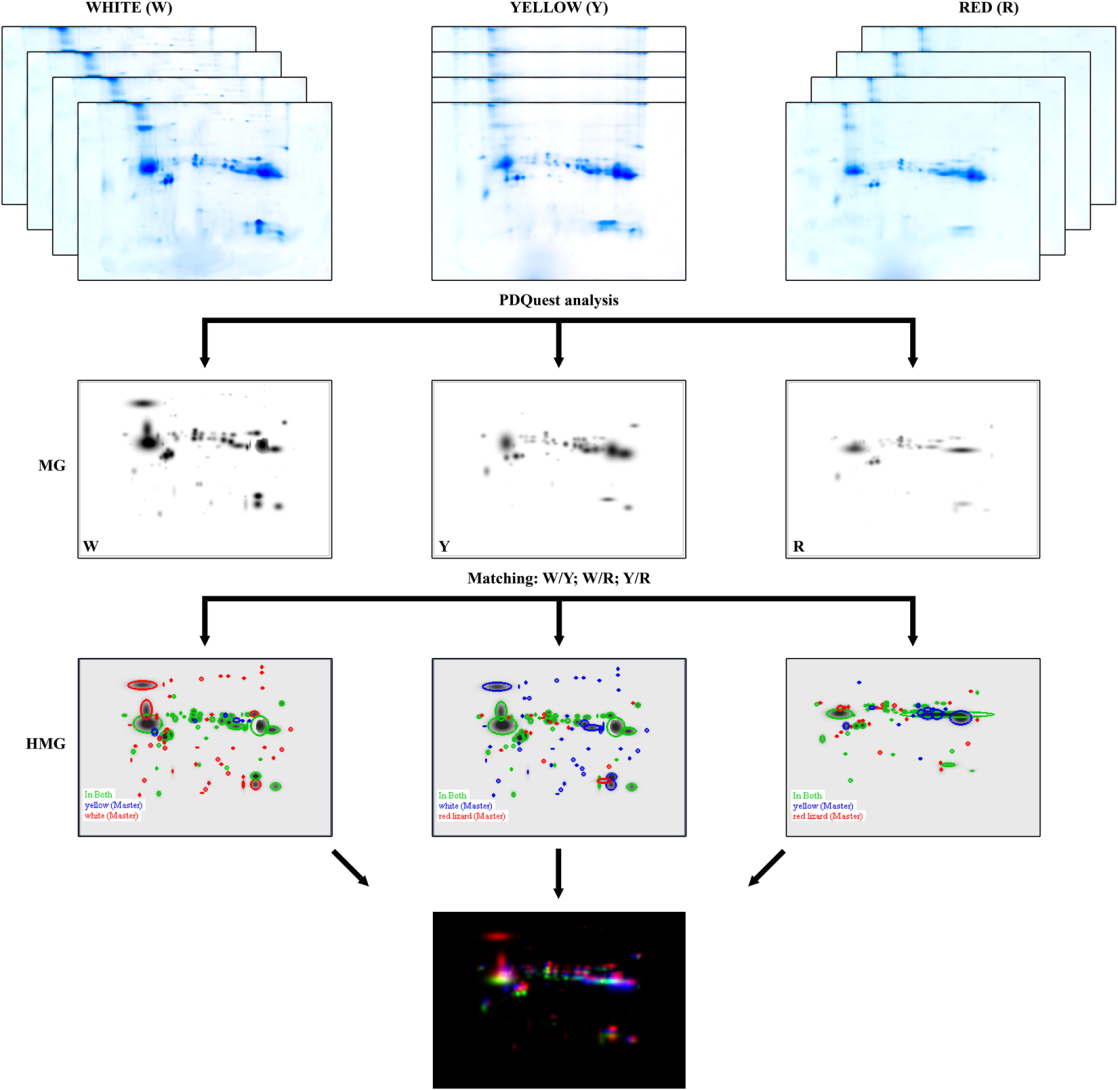
Scheme of the two-dimensional electrophoresis analysis. Top: scanned images of the four gels obtained from each morph sample (original images are available as Supplementary Information); MG: master gel after PD Quest (Biorad) elaboration, representing the virtual gel associated to each morph (from left to right: white, yellow, red); HMG: high master gel obtained by the comparison of each MG pair (from left to right: W vs Y, W vs R, and Y vs R); bottom: combined high master gel (CHMG) obtained by superimposing the three HMGs to highlight those spots unique to each morph: red = W, green = Y, and blue = R.

The original gels from 2-DE are available as Supplementary Information. The master gels of W, Y and R morphs are shown in in the mid-line of Fig. 1, left to right, respectively. The mean spot number in the gels was 84, 53, and 55 for morphs W, Y, and R, respectively. The number of spots in W morph was about 1.5 fold higher than in R and Y.

The comparison of master gel patterns allowed to generate three new virtual images indicated as High Master Gels (HMG; Fig. 1) that evidenced these differences. In particular, the HMG generated by matching Y against W (Fig. 1) revealed that 47 (68.6%) spots were common to both phenotypes; 37 (27.0%) were unique of W and 6 (4.4%) exclusive of Y. Likewise, the HMG produced when R was matched against W (Fig. 1) showed that 40 (57.6%) spots were common to both phenotypes; 44 (31.7%) were exclusive of W and 15 (10.8%) of R. Finally, the HMG obtained from the comparison of Y and R master gels (Fig. 1) showed that these morphs had 32 (59.3%) spots in common; 21 (19.4%) were unique of Y and 23 (21.3%) of R. Taking advantage of the similarity among patterns, the three HMGs were correlated to each other (Y *vs* W; R *vs* W and R *vs* Y) to understand which were the spots common to all morphs and which unique to each of them. The same procedure mentioned above allowed the creation of the final virtual image indicated as CHMG (Fig. 1), comprehensive of all matched spots derived from the three HMGs.

### Mass spectrometry (MS) analysis of differential proteins

**Figure 2:**
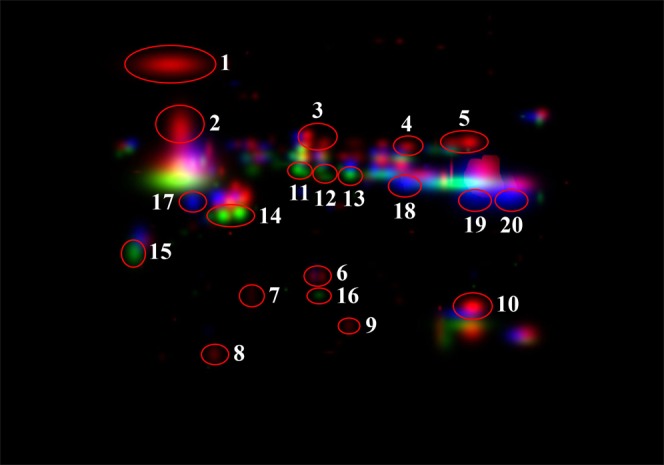
Position on the CHMG of the 20 excised spots finally used in mass spectrometry analysis. Numbers 1–10 belong to W, 11–16 to Y, 17–20 to R.

As it can be seen from the magnified picture of CHMG (Fig. 2), a red, green, and blue colour was assigned by the software to spots exclusive of morph W, Y, and R, respectively. Among the spots peculiar of W morph, ten (numbered 1 to 10 in Fig. 2) were apparently not overlapping with others. The same for six spots unique to morph Y (numbered 11 to 16 in Fig. 2) and four unique to morph R (numbered 17 to 20 in Fig. 2). All these spots were carefully excised from the gel, destained, digested with trypsin and peptides submitted to MS analysis.

A scheme illustrating the peptide-spectrum matching results on the MS data is shown in Table 1. The low abundance of proteinaceous material under spot 2, 7, 8, 9, and 16 most likely determined the poor quality of their MS signals, which prevented any identification attempt. These spots were then excluded from the subsequent spectrum-to-spectrum comparisons. Seven spots (3, 5, 6, 12–14, 17) did not produce any match, the remnants eight gave a total of 14 identified peptides, seven unique to W, three to Y, and four to R. Six identified peptides matched proteins known to be linked to skin colour (Tables 1 and S3 in^36^). The lack of multiple peptide matches against a single protein prevented any identification at protein level.

**Table 1:** List of the identified peptides using the database from Table S3 in^36^.

morph	Spot	peptide	error	score	FDR	accession	gene	description	Colour link
W	1	R.RCRCFR.R	−0.063	9.160	0.000	O75443	TECTA2	tectorin alpha gene 2	
		R.FQGNLWK.T	0.057	9.082	0.000	G1SEM4	ADA	adenosine deaminase	Purine metabolism, Tyrosine
		K.DYVNDLKDSYGQEWTR.Y	−0.085	9.053	0.000	P54707	ATP12A	ATPase H+/K+ transporting nongastric alpha polypeptide	Purine metabolism
	3	Unknown							
	4	K.YNIEEEGTWR.R	−0.030	8.901	0.000	F6TWE8	OBSCN	obscurin cytoskeletal calmodulin and titin-interacting	Purine binding, Tyrosine
	5	Unknown							
	6	Unknown							
	10	K.TPEGTLPR.L	0.228	8.640	0.000	A0A2R9A5X2	AXDND1	axonemal dynein light chain domain containing 1	
		K.RQMHKPIK.V	−0.447	7.622	0.000	W5UKP0	CYLD	cylindromatosis	
		K.GTDPQVR.Y	0.249	7.618	0.000	I3J9Y8	PARP9	poly (ADP-ribose) polymerase family member 9	
Y	11	K.VLSVHPWNRPSLQDCLAHPWLQDAYLMKLR.R	−0.454	9.182	0.000	G3TQN9	SPEG	SPEG complex locus	Purine binding
	12	Unknown							
	13	Unknown							
	14	Unknown							
	15	R.LTVGTRPDGLPDERWCFR.V	0.143	7.593	0.000	A0A2U4C2P6	TRPV2	transient receptor potential cation channel subfamily V member 2	
		K.TWTSFLSGVNIQIVGDDLTVTNLK.R	−0.262	7.512	0.000	Q1KYT0|ENO3	ENO3	enolase 3	Iridophore, Purine
R	17	Unknown							
	18	R.DIPKGIR.Q	0.167	7.609	0.000	A0A096NX44	WFDC3	WAP four-disulfide core domain 3	
	19	K.DINTFVHGNRHHITAICGDENGSPYGGNLR.I	−0.321	8.038	0.000	Q8WN63	ANG	angiogenin ribonuclease RNase A family 5	
	20	K.LSASSEASEVDKKEK.S	−0.384	8.373	0.000	A0A2K6EX08	DTX3L	deltex 3-like	
		K.GGGAPK.T	−0.348	7.987	0.000	A0A2K5QEN8	MYO18B	myosin XVIIIB	Purine binding

Error = difference between the measured and calculated parental ion mass (Da); score = MSGF+ spectrum E-value (−log_10_ transformed); FDR = false detection rate at the peptide level; accession = uniprotKB accession; gene = gene name as reported in Table S3^36^; description = protein description as reported in Table S3^36^; colour link = previous link to colour as reported in Table S3^36^. Spots 2, 7, 8, 9, and 16 are not shown due to poor quality spectra.

**Figure 3:**
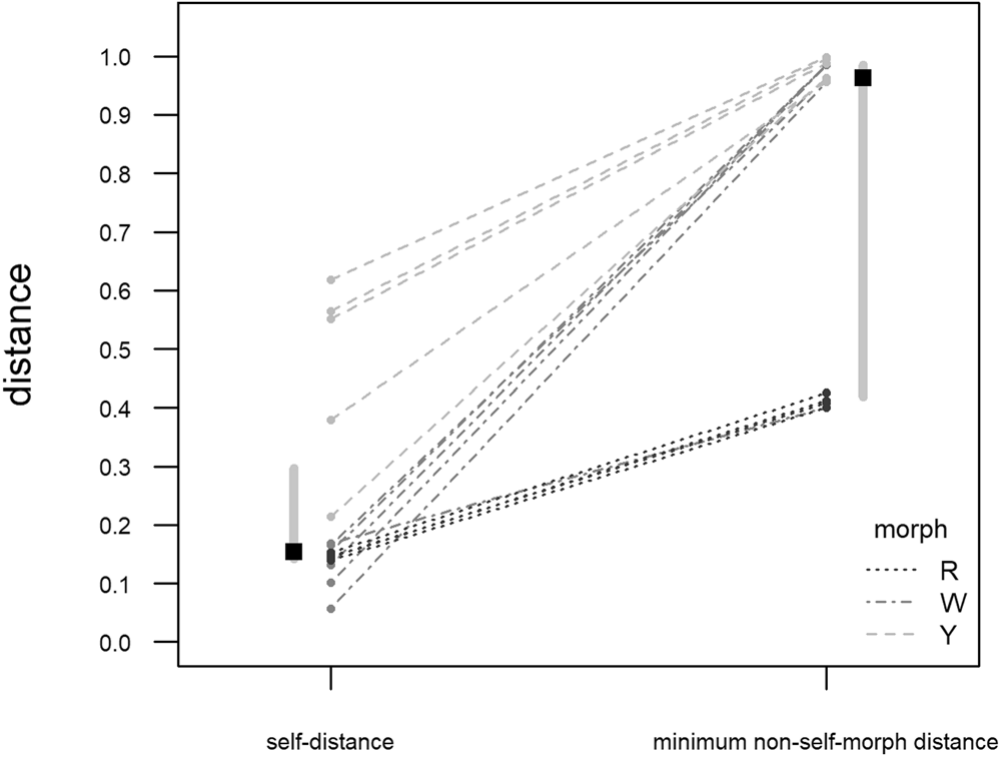
Comparison between the spectrum-to-spectrum distance of each analysed spot from itself (self-distance) and from the most similar spot among the ones belonging to a different morph (minimum non-self-morph distance). Values on the ordinate are cosine distance. Grey dots = observed distance value; dashed lines are used to link each self-distance to the corresponding non-self-morph. Black squares represent the medians of self- and non-self-morph distances; vertical grey bars show the interquartile range for each distance group.

The spectrum-to-spectrum comparison showed that there were no two identical spectra (105 pairwise comparison; Table 2) and highlighted the distinctness of the morph-specific spots (Fig. 3): the median “minimum non-self distance” was 0.963 (inter-quartile range = 0.567), while the median “self-distance” was 0.154 (inter-quartile range = 0.155). The difference is highly significant (Wilcoxon signed rank test: W = 0.000; P < 3.052·10^−5^; n = 15).

**Table 2:** Pairwise distance matrix obtained from the spectrum-to-spectrum comparison of the spots that gave reliable spectra.

spot	#01	#03	#04	#05	#06	#10	#11	#12	#13	#14	#15	#17	#18	#19	#20
#01	*0.06*	**0.95**	0.96	0.99	1.00	0.96	0.98	0.96	1.00	1.00	1.00	1.00	1.00	1.00	1.00
#03	**0.95**	*0.13*	**0.95**	0.98	1.00	0.96	0.99	0.99	0.99	1.00	1.00	1.00	1.00	1.00	1.00
#04	0.96	**0.95**	*0.15*	0.98	1.00	**0.95**	0.99	0.99	0.99	1.00	1.00	1.00	1.00	1.00	1.00
#05	0.99	0.98	0.98	*0.10*	1.00	**0.97**	0.99	0.99	0.99	1.00	1.00	1.00	1.00	1.00	1.00
#06	1.00	1.00	1.00	1.00	*0.17*	1.00	1.00	1.00	1.00	1.00	1.00	**0.40**	0.41	0.41	0.43
#10	0.96	0.96	**0.95**	0.97	1.00	*0.17*	0.96	0.97	0.99	1.00	1.00	1.00	1.00	1.00	1.00
#11	0.98	0.99	0.99	0.99	1.00	**0.96**	*0.21*	0.98	0.99	1.00	1.00	1.00	1.00	1.00	1.00
#12	**0.96**	0.99	0.99	0.99	1.00	0.97	0.98	*0.38*	0.99	1.00	1.00	1.00	1.00	1.00	1.00
#13	1.00	**0.99**	**0.99**	**0.99**	1.00	**0.99**	**0.99**	**0.99**	*0.55*	1.00	**0.99**	1.00	1.00	1.00	1.00
#14	1.00	1.00	1.00	1.00	1.00	1.00	1.00	1.00	1.00	*0.62*	**0.99**	1.00	1.00	1.00	1.00
#15	1.00	1.00	1.00	1.00	1.00	1.00	1.00	1.00	**0.99**	**0.99**	*0.56*	1.00	1.00	1.00	1.00
#17	1.00	1.00	1.00	1.00	0.40	1.00	1.00	1.00	1.00	1.00	1.00	*0.14*	**0.41**	0.42	0.42
#18	1.00	1.00	1.00	1.00	0.41	1.00	1.00	1.00	1.00	1.00	1.00	0.41	*0.15*	**0.40**	0.41
#19	1.00	1.00	1.00	1.00	0.41	1.00	1.00	1.00	1.00	1.00	1.00	0.42	**0.40**	*0.14*	0.42
#20	1.00	1.00	1.00	1.00	0.43	1.00	1.00	1.00	1.00	1.00	1.00	0.42	**0.41**	0.42	*0.15*

Values are cosine distance between spectra from each spot pair. The diagonal represents the “self-distance” values for each spot (italicized); in each row, the values corresponding to the “minimum non-self distance” for each spot are bolded.

## Discussion

The proteinaceous content of the femoral pore secretions of *Podarcis muralis* appears well-differentiated among the three pure colour morphs, being W the richest in term of spot number (84 distinct spots), followed by R (55) and Y (53), which have similar scores. Out of 84, 15, 6, and 4 spots uniquely occur in W, Y and R, respectively. Even assuming these distinct spots could arise from protein under-expression rather than a true absence, the differences in the observed patterns are such as to allow easily discriminate each colour morph by 2-DE profiles alone (Fig. 1). Moreover, though not allowing protein identification, the MS data confirm that the differential spots hold unique peptides (Table 1; Fig. 3), making the 2-DE outcome further supported.

A similar match between chemical profile and colour morph has been already found in this species for the lipophilic part of the femoral gland secretions^32^. Whereas lipids are well-recognized chemical signals in lizard^83^, and relatively few studies have explicitly related proteins to inter-individual chemical communication^93,111–115^, the coherence between outcomes of the two studies on lipid and protein may be the result of a correlative effect: proteins simply form the non-informative matrix where lipids lie^62,116^, and, accordingly, any variation in lipid composition will be indirectly reflected in the protein one. This interpretation has a weak experimental support, though. The difference in lipid profiles is not as strong as that of proteins. Pellitteri-Rosa *et al*.^32^ found R-morph having relatively more tocopherol and less furanone than W, but only W showed a significant difference in the overall profile, and the attempt to classify morph on the lipids basis did not score well. This weakness can be explained considering that samples for the lipid study came from three distinct populations (no information are available about the site × morph frequency in the sample) over a period of two months (April to May). As both population and season can affect the composition and amount of the lipid fraction^67,90,92,117–121^, potentially in a morph-specific way^43,44,122^, an unbalanced sampling of morphs by period and population could have biased results. On the opposite, the observed differences in the protein pattern cannot be imputed to population, timing, or to sampling bias, since all sampled lizards came from the same site, were collected on the same day, at the peak of the breeding season^43,86^, and the pooled secretions were obtained by balancing the contribution of each donor (see Material and Methods). So, the stronger and more robust results from protein comparison are in contrast with what would be expected under a correlative hypothesis, which, at most, would have predicted the opposite, i.e., a stronger relation with lipids.

From a theoretical point of view, proteins look like a more probable candidate than lipids to convey information about morph, given morphs to represent equally adapted traits combinations^5,7,9^, genetically hereditable^100^, and unrelated to individual quality^8,11^, i.e., individual quality is still part of the story, but within each morph. Most lipids (or their precursors) from femoral glands cannot be synthetized *ex-novo* by lizards^53,83,88^. Rather, they are acquired from the environment, and impose a cost to their use in communication: this is exactly what a reliable quality signal does^123^, and evidences of such function have been already collected^53,81,90–92,124,125^. On the other side, proteins own an undoubted morph-specific profile, have a direct link with genes, do not impose an actual cost to the emitter (*sensu* Zahavi and Zahavi^123^), and can be detected by lizards^69,93,126^ thanks to the vomeronasal organ and taste. Altogether, these properties give the proteins the potential of being used as proxy for colour morph, as a part of a more complex chemical badge^73,97,127^. Future studies about the design of lizard chemical communication should hence adopt an integrated approach that simultaneously considers both chemical fractions of the signal, disentangle the unique information they carry, and investigate how they influence each other.

Finding a morph-specific pattern in proteins secreted by femoral gland has important consequences for the understanding of intra-specific interactions among free-ranging individuals of both sexes. Proteins are not volatile. When they are exploited as semiochemicals in terrestrial animals, they are usually in water solution (e.g., urine^111,128^) or directly transferred on the receiver chemoreceptive surfaces during close interactions (e.g., plethodontid salamanders^129^). In lizards, femoral gland secretions are typically left on dry substrates^62,130^, and the only way they can be detected is through the direct inspection, i.e., tongue-flicking^54,59^. Nevertheless, proteins are long-lasting stable marks (1-d electrophoresis of three-years-old samples gave the same results as freshly collected ones; Mangiacotti *et al*., unpubl.), and are among the most suitable signals in territorial contests^131^. Indeed, typically territorial species are able to recognize familiars on a chemical basis^50,74–76,132^, and also to build a spatial map of scent marking points^133^. In a CP system, assessing the morph identity of a potential rival or mate without (or before) seeing it (i.e., before the visual modality can be activated) may give a great advantage in decision-making and allows better tuning intraspecific interaction^12,39^. Indeed, non-random mating has been recognized as a key mechanism contributing to CP maintenance^9^, and it has been reported also for the common wall lizard^42,45^, where both male-male competition^109,110,134^ and female flexible choice^45,135^ seem to be at work. Combined with female preference for chemical rather than visual *stimuli*^135^, the occurrence of a dual modality (visual and chemical) of morph-specific signals gains even more importance.

Unfortunately, the identification of the involved proteins has not been achieved, thus preventing us to shed light on the mechanism behind morph chemical signalling. The lack of a specific and targeted database to match against MS spectra and the absence of previous knowledge about the composition of proteins from lizard femoral glands^63,73^ are probably the reasons for this trouble. The chosen database could have been hypothetically suitable, in that it pertained the skin gene expressions of a polymorphic lizard^36^, but retrieved sequences came from phylogenetic distant species, maybe too distant to give better results. Nonetheless it allowed the identification of some differential peptides, which, together with 2-DE and spectrum-to-spectrum comparison, is enough to fix that morph-specific proteins are actually present, which was the primary study aim. Now, more targeted work is needed to obtain a list of secreted proteins, to understand their role, also in relation to the lipophilic fraction, and the underlying mechanisms, in order to attempt a more multi-modal approach to animal communication.

The question of whether all the involved proteins (or only a few of them) have to do with differences among morphs’ chemical profiles rather than to other individual traits, as well as if lizards are actually able to discriminate morphs based on the protein fraction alone need to be proven by further molecular investigations and behavioural tests. The results of this pilot study just add a further step towards the comprehension of the mechanisms by which chemical and visual signalling cooperate in driving lizards’ communication and CP maintenance.

## Materials and Methods

### Sample collection

A total of 30 adult males (snout-to-vent length: mean = 64.7 mm; range: 59.0–71.0 mm^136^) of the common wall lizard *Podarcis muralis* have been considered in this study. Lizards were captured by noosing, which did not cause the animal avoidable pain, suffering, distress or lasting harm^137^. To minimize sample heterogeneity, all lizards were captured at the same site (Castelseprio, Lombardy, Italy: 45.73°N, 8.86°E, 358 m a.s.l.). Further, to avoid uncontrolled seasonal effects^86^, captures were concentrated on a single day (3^rd^ April 2017), at the beginning of the breeding season, when glandular activity is at its maximum^86^ and males of the three morphs show comparable testosterone levels^43^. According to the differences in their ventral coloration (see Fig. 1 in^99^), lizards were assigned to one of the three pure morphs: white (W), yellow (Y), and red (R). Only lizards showing pure morphs were considered^99^. The final sample included ten individuals for each morph.

Femoral gland secretions were obtained from each individual by applying a gentle pressure around the thighs with the help of a small steel spatula, and collecting the protruding plugs directly into glass vials^73^. Lizards were then released at the capture point. Vials were transferred to the laboratory and samples preserved at −20 °C until analyses^73^.

No lizards were killed or injured during the study. Permits for capturing and handling lizards were granted by the Italian Ministry of Environment (Prot. Aut. PNM-2015-0010423; PNM-2016-0002154), who also approved sampling collection (which was not invasive and did not cause damage to any animal tissues).

### Extraction and quantification of proteins

Secretions of male lizards femoral glands were pooled according to the morph. Proteins were extracted from waxy secretions through a defatting procedure^73^. In brief, 200 µL of n-hexane were added to samples (an average of 1–2 mg of proteins), incubated at room temperature for 2 h and, after centrifugation (14,000 rpm for 10 min), proteins were isolated as a pellet. The procedure was repeated three times and proteins were finally air-dried. Protein pellets were then dissolved in 200 µL of 10 mM PBS buffer pH 7.4, containing 137 mM NaCl and 2.7 mM KCl. Their exact quantification was achieved by applying the Bicinchoninic Acid (BCA) assay using bovine serum albumin (BSA) as the standard protein for the production of the calibration curve (in the range of concentration between 5 and 25 μg/mL). At this point, aliquots belonging to the individuals of the same group and containing a similar quantity of proteins were pooled, according to the morph. The protein concentration was about 2,5 mg/mL for each group of individuals and the total amount of proteins was about 1.0 mg/group.

### Two-dimensional electrophoresis

#### Protocol set up

Samples were prepared by dissolving about 150 μg of proteins in 125 μL of rehydration buffer (8 M urea, 4% CHAPS (w/v), 65 mM DTE, 0.8% carrier ampholytes (v/v), 0.5% bromophenol blue). As 2-DE was never carried out before on proteins from lizard femoral glands, some preliminary attempts were made in order to attain a satisfactory outcome. Notably, the first dimension (isoelectric focusing - IEF) was run with linear and non-linear IPG strips, having the same pH range (pH 3–10; Amersham Biosciences, UK); for the second dimension the porosity of the SDS polyacrylamide gel was alternatively set to 12.5% or 15%.

Samples were first loaded onto 7 cm IPG strips, which were rehydrated without applying voltage for 1 h at 20 °C. IEF was carried out at 15 °C using an Ettan IPGphor system (Amersham Biosciences), programmed with the following voltage gradient: 30 V for 8 h, 120 V for 1 h, 500 V for 0.5 h, 1000 V for 0.5 h and 5000 V until a total of 25–27 kV/h was reached. Reduction/alkylation steps were applied between the first and the second dimension. The focused IPG strips were incubated for 15 min at room temperature in 6 M urea, 2% (w/v) SDS, 50 mM Tris pH 6.8, glycerol 30%, containing 2% (w/v) DTE, followed by a second incubation of 15 min in the same buffer containing 2.5% (w/v) iodoacetamide and 0.5% bromophenol blue. At the end of the IEF step, strips were hold in place with 0.4% low melting temperature agarose and loaded onto 8 × 6 cm slabs, 12.5% or 15% SDS polyacrylamide gels^73^. Electrophoresis was carried out at a constant current of 10 mA per gel in a PROTEAN II xi 2-D Cell equipment Bio-Rad (Berkeley, California), until the buffer frontline was 1 mm from the bottom of the gels. The 2-DE gels were stained with “Blue silver” (colloidal Coomassie G-250 staining)^138^. To minimize the technical mistakes connected with sample manipulation, experimental steps concerning sample preparation and electrophoretic runs were performed “in parallel” on all samples.

The visual inspection of the preliminary gels highlighted: (i) an unexpected overcrowding of spots being evident at the bottom of the slabs when using 12.5% porosity in second dimension; (ii) a lateral compression of spots, leaving a poorly coloured central area, when IEF used non-linear IPG strips. The best outcome, which minimized spot overlap and blank areas, was attained with linear strip and 15% porosity. Given the good resolution of spots, 2-DE analyses were performed in quadruplicate for each group (W, Y, R) using the above settings, to produce the 12 gels used in the final comparison (Fig. 1).

#### Gel analysis

Digital images of stained gels were acquired using the VersaDoc Imaging Model 3000 (BioRad) and then subjected to quali/quantitative analysis using the PD Quest (BioRad) version 8.0.1 software. Spot detection was achieved using the spot detection wizard tool after defining and saving a set of detection parameters. After spot detection, the original gel scans were filtered and smoothed to clarify spots, remove vertical and horizontal streaks and remove speckles. Three images were created from the process: the original raw 2-D scan, the Filtered image and the Gaussian image. A match set for each group was then created for comparison after the gel images had been aligned and automatically overlaid. If a spot was saturated, irregularly shaped, or otherwise of poor quality, then the Gaussian modelling was unable to accurately determine quantity. In these cases, the spot was defined in the filtered image using the spot boundary tools. Thus, for each group, a virtual image was produced which included protein spots only if present at least in two out of the three best gels. This is indicated as “master gel”.

### Mass spectrometry analysis

#### *In situ* enzymatic digestion

The selected spots (Fig. 2) were carefully excised from the gel, placed into Eppendorf tubes and broken into small pieces. This material was then washed twice with aliquots (200 μL) of 100 mM ammonium bicarbonate buffer pH 7.8, 50% acetonitrile (ACN) and kept under stirring overnight, until complete destaining. Gels were dehydrated by addition of ACN (100 μL). After removal of the organic solvent, reduction was performed by addition of 50 μL of 10 mM Dithiothreitol (DTT) solution (40 min at 37 °C). DTT was replaced with 50 μL of 55 mM iodoacetamide for 45 min at 56 °C. This solution was removed and the gel pieces were washed twice with 200 μL of 100 mM ammonium bicarbonate for 10 min, while vortexing. The wash solution was removed and gel dehydrated by addition of 200 μL of ACN until the gel pieces became an opaque-white color. ACN was finally removed and gel pieces were dried under vacuum. Gels were rehydrated by addition of 75 μL of 100 mM ammonium bicarbonate buffer pH 7.8, containing 20 ng/μL sequencing grade trypsin (Promega, Madison, WI, USA) and digestion was performed overnight at 37 °C. Following enzymatic digestion, the resultant peptides were extracted sequentially from gel matrix by a three-step treatment (each step at 37 °C for 15 min) with 50 μL of 50% ACN in water, 5% trifluoroacetic acid (TFA) and finally with 50 μL of 100% ACN. Each extraction involved 10 min of stirring followed by centrifugation and removal of the supernatant. The original supernatant and those obtained from sequential extractions were pooled, dried and stored at −80 °C until mass spectrometric analysis. At the moment of use, the peptide mixture was solubilized in 100 μL of 0.1% formic acid (FA) for MS analyses.

#### LC-MS/MS

All analyses were carried out with a liquid chromatography-mass spectrometry (LC-MS, Thermo Finnigan, San Jose, CA, USA) system consisting of a thermostated column oven Surveyor autosampler controlled at 25 °C, a quaternary gradient Surveyor MS pump equipped with a diode array detector, and an Linear Trap Quadrupole (LTQ) mass spectrometer with electrospray ionization ion source controlled by Xcalibur software 1.4. Analytes were separated by reverse phase high performance liquid chromatography (RP-HPLC) on a Jupiter (Phenomenex, Torrance, CA,USA) C_18_ column (150 × 2 mm, 4 μm, 90 Å particle size) using a linear gradient (2–60% solvent B in 60 min) in which solvent A consisted of 0.1% aqueous FA and solvent B consisted of ACN containing 0.1% FA. Flow-rate was 0.2 mL/min. Mass spectra were generated in positive ion mode under constant instrumental conditions: source voltage 5.0 kV, capillary voltage 46 V, sheath gas flow 40 (arbitrary units), auxiliary gas flow 10 (arbitrary units), sweep gas flow 1 (arbitrary units), capillary temperature 200 °C, tube lens voltage–105 V. MS/MS spectra, obtained by CID studies in the linear ion trap, were performed with an isolation width of 3 Th m/z, the activation amplitude was 35% of ejection RF amplitude that corresponds to 1.58 V^139^.

#### Protein identification

Protein identification was attempted using a peptide-spectrum matching (PSM) approach^140,141^, as implemented in the MS-GF+ v2018.07.17 software^142–145^. According to the instrument sensibility, digestion protocols^140,141^, and general guidelines^142^, the algorithm settings were as follows: tolerance, 0.5 Da; charge range, 1–6+; range of peptide length, 6–35; isotope error 0–2 Da; cleavage, semi-tryptic; post translational modification, fix carbamidomethylation of cysteine^140,146,147^. The database choice is a crucial step in PSM, and, unfortunately the study species and the peculiarity of the protein samples prevented the extraction of an actually reliable dataset from the usual repositories^148^. So, an *ad hoc* database was built by taking advantage from the paper by McLean *et al*.^36^, where a list of differentially expressed genes at the skin level was made available for the colour morphs of the tawny dragon, *Ctenophorus decresii* (Table S3 in^36^). Even if the tawny dragon (Order Squamata, Fam. Agamidae) is not phylogenetically close linked to the common wall lizard (Order Squamata, Fam. Lacertidae), McLean’s and our study share these common main points: (i) they both involve polymorphic lizards; (ii) they both involve tissues with an epidermal origin; (iv) proteins conveying information about colour could derive from, or be related to, the same set of genes involved in skin colouration. The UniProt Knowledgebase release 2018_07^149^ was then surveyed for the 458 unique gene names available in Table S3^36^, and the so-obtained entries were filtered out to match the vertebrate taxon. Further, to account for any contamination^147^, mammalian trypsin and human keratin sequences, also retrieved from UniProt, were added to the previous database. The final dataset counted 59,622 unique sequences.

To maximize power, PSM was run as a two-stage process^150^ with target-decoy approach. All the candidate proteins identified in the first stage (target or decoy) were used in the second stage to refine identification^151^, adjusting the proportion of target/decoy sequences to reach an unbiased estimation of false detection rate (FDR)^151–153^. Decoy sequences were obtained by reversing the target ones in both stages. FDR was calculated at the peptide level as n_decoy_/n_target_ for a given spectrum E-value, which was used as score^151^. Before FDR computation, the list of identified spectra was purged from all the spectra (i) simultaneously matching target and decoy sequences, (ii) corresponding to peptides with semi-tryptic cleavage, and iii) having more than two irregular cleavage^151^. Only spectra with FDR ≤ 0.01 were considered. A protein was considered identified if more than two different peptides match the same protein.

To further assess the effective distinctness of morph-specific spots, a pairwise spectrum-to-spectrum comparison was performed^154–156^. The set of spectra from each MS run was compared to all the others belonging to a different morph, and the cosine distance computed^155^. The minimum of this distances for each spot (minimum non-self-distance) was retained and compared to the one computed between each spot and itself (self-distance). A Wilcoxon signed rank test (one tail, with exact P estimation) was then used to assess if self-distance was significantly smaller than minimum non-self distance^157^, and to exclude spots identity.

All the above operations were implemented in R v3.5.0^158^, using the packages mzID^159^, Biostrings^160^, stringr^161^, functions by Rieder *et al*.^155^, and *ad hoc* functions (available upon request) to prepare database and call external software (MSGF+).

## Supplementary information

Images of the original 2D-electrophoresis gels

## Data Availability

The scans of the best three 2-DE gels and MS raw data used in this study are available in Zenodo archive (10.5281/zenodo.1460606).
