# Supplementary material for: Morph-specific protein patterns in the femoral gland secretions of a colour polymorphic lizard
Source: Sci Rep. 2019 Jun 10;9:8412. doi: 10.1038/s41598-019-44889-7 (PMC6557888; doi:10.1038/s41598-019-44889-7)
Supplement: Supplementary file 1 — Images of the original 2D-electrophoresis gels [file 41598_2019_44889_MOESM1_ESM.pdf]

Morph-specific protein patterns in the femoral gland secretions of a colour polymorphic lizard

Marco Mangiacotti, Marco Fumagalli, Maddalena Cagnone, Simona Viglio, Anna Maria Bardoni, Stefano Scali, Roberto Sacchi

SUPPLEMENTARY INFORMATION

Original 2DE gels. Here it follows the image list of the gels from the two-dimensional electrophoresis as they were originally acquired by VersaDoc Imaging Model 3000 (BioRad). Images were not manipulated, nor contrast and luminosity were altered. The same settings were used in each acquisition. For each colour-morph, the three best replicates actually used in the analysis are reported. Further, for each morph series, an inset with pH and weight scales has been added.

Red morph	replicate #
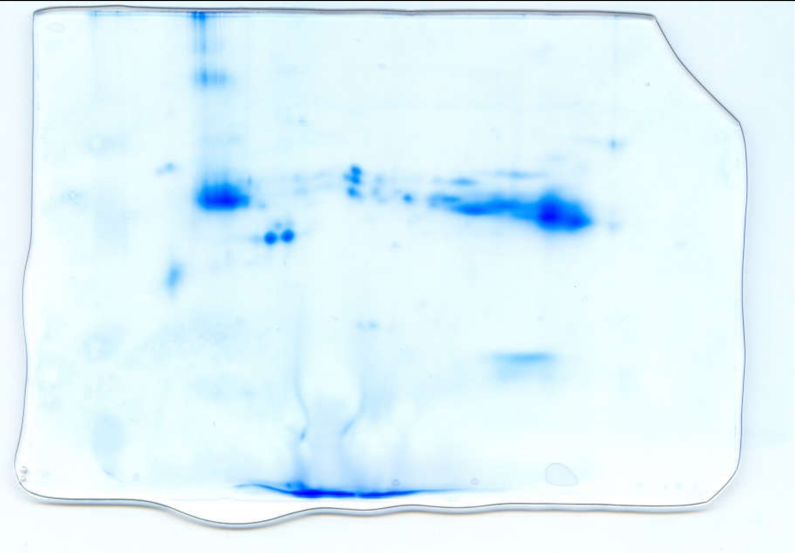	1
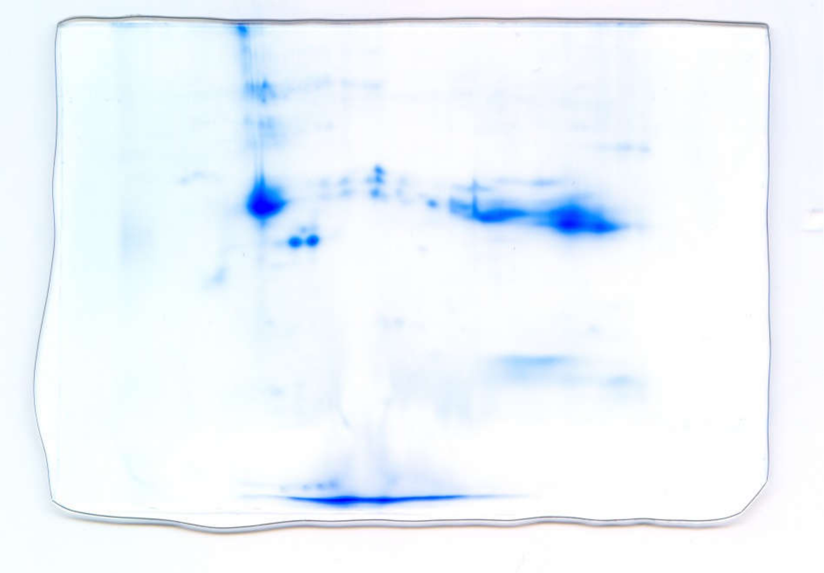	2
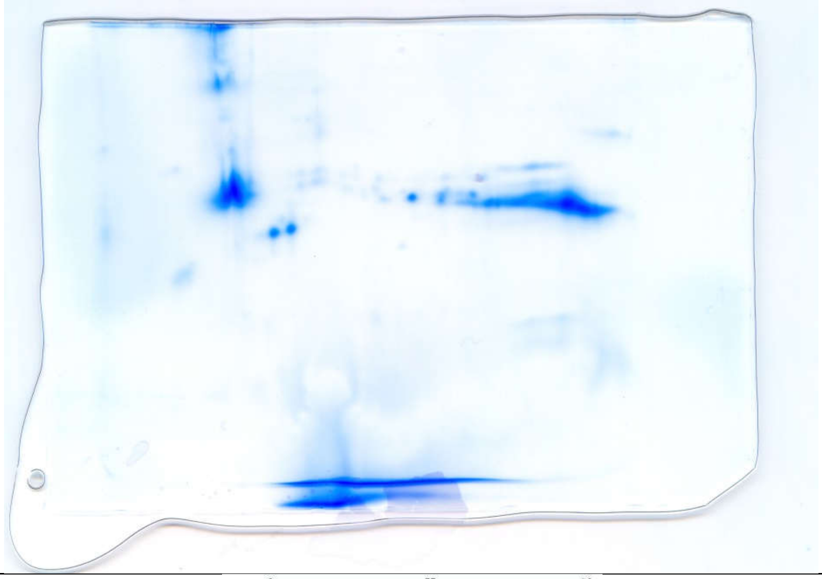	3
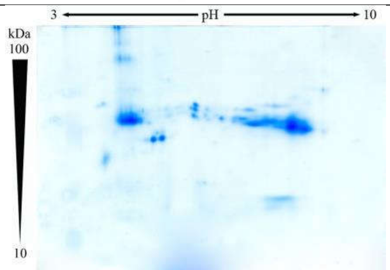	scale

White morph	replicate #
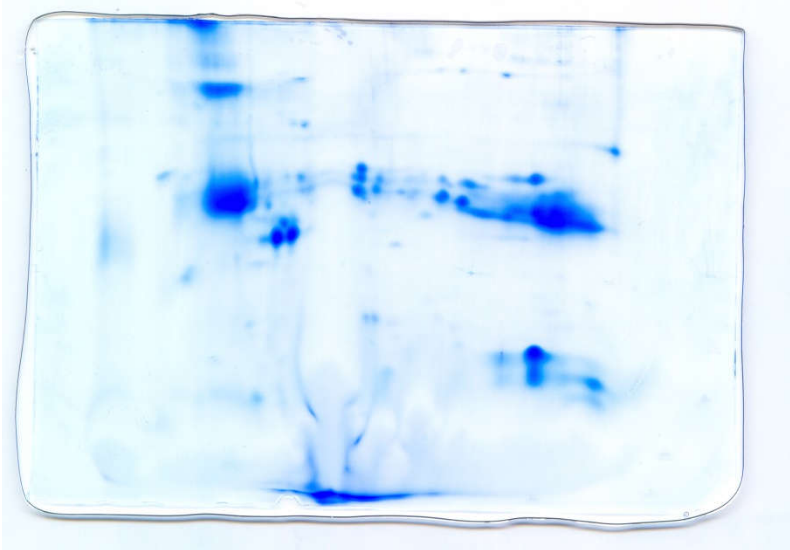	1
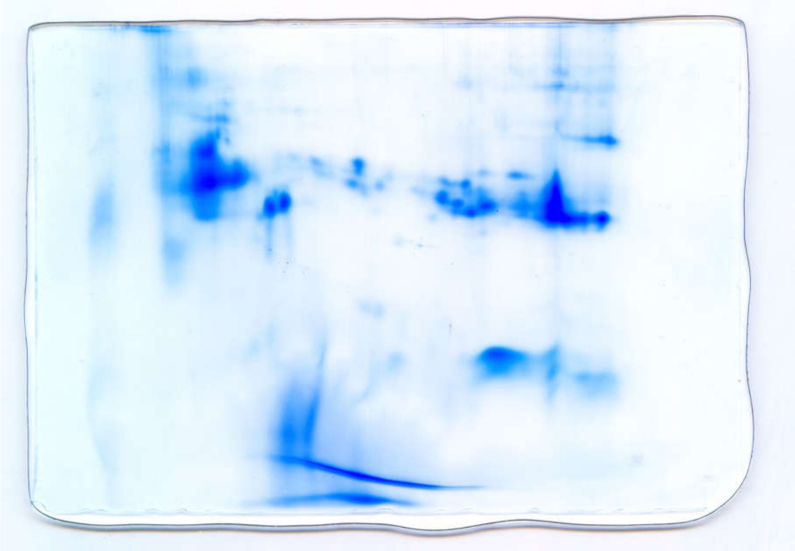	2
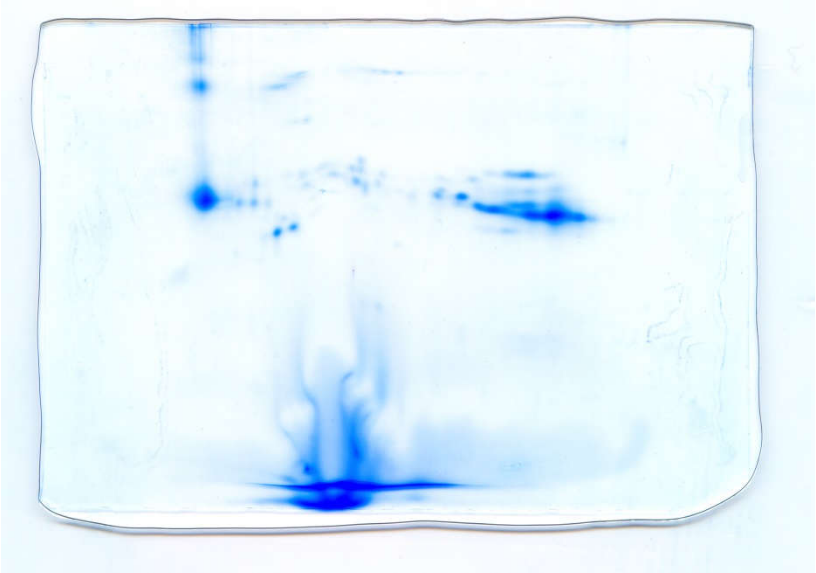	3
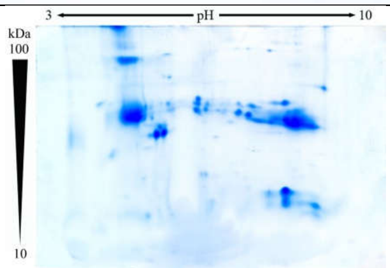	scale

Yellow morph	replicate #
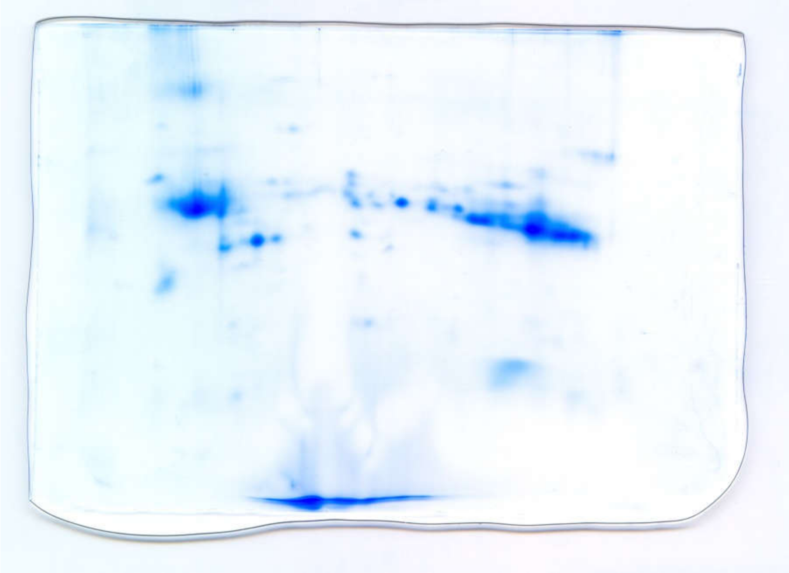	1
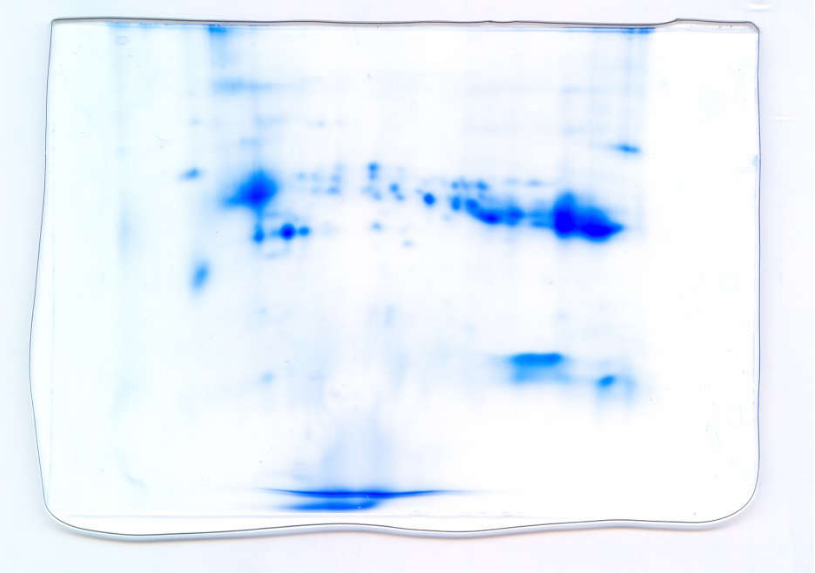	2
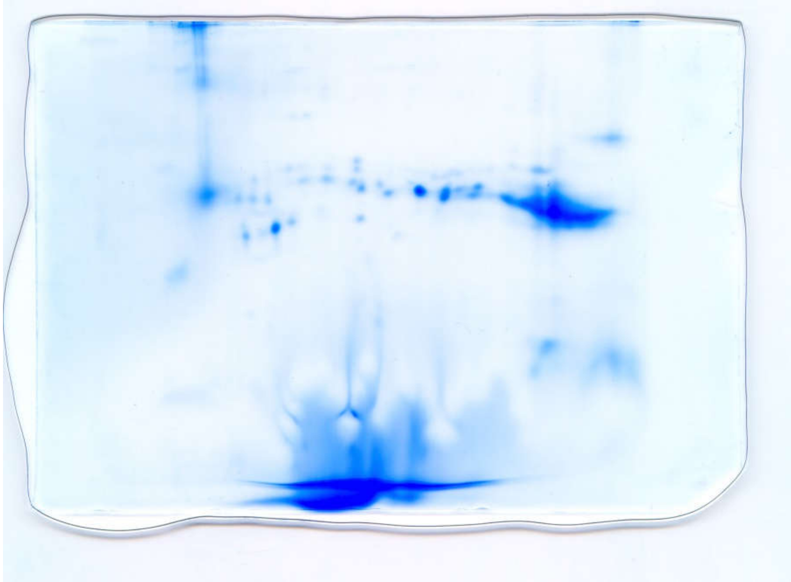	3
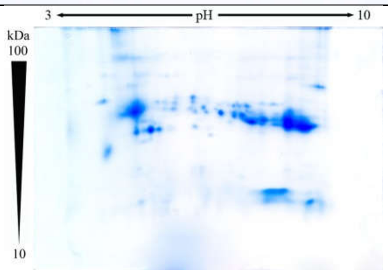	scale
